# Psychological Capital of Entrepreneur Teams and Human Resource Development

**DOI:** 10.3389/fpsyg.2020.00274

**Published:** 2020-03-24

**Authors:** Jun-Jun Tang

**Affiliations:** ^1^College of Economics and Administration, Nanjing University of Aeronautics and Astronautics, Nanjing, China; ^2^Huaiyin Institute of Technology, Huaian, China

**Keywords:** psychological capital, human resource development, future research, entreprenership, human capital

## Abstract

The ability of an organization to materialize its objectives largely depends on the psychological and physical participation of the employees and staff members. The theory of psychological capital focuses on how an individual is changing or evolving, rather than who that individual is. Thus, the concept of PsyCap should be tightly tied and discussed with human resource development (HRD), both from practical and theoretical lens. This paper sets to discuss the strategic and entrepreneurial associations between PsyCap and HRD. The goal is to shed light on potential practical designs and theoretical improvement in the future.

## Background

The human resource of an entrepreneurial venture plays a crucial role in determining its short-term survival and long-term success, thereby necessitating an in-depth analysis of the underlying influencing factors. The ability of an organization to materialize its objectives largely depends on the psychological and physical participation of the employees and staff members. A higher degree of participation and voluntary commitment to organizational goals can lead to higher competitive advantages, which is crucial for the long-term growth of the company (Cavus and Gokcen, 2015). The organizations, in their pursuit of improving performance and operational efficacy, are looking forward to achieving positive organizational behavior, through developing positive psychological states among the team members.

In this context, the concept of ‘psychological capital,’ also commonly known as “PsyCap” can be discussed, and its influence on teams and human resource development (HRD) can be reviewed. Luthans et al. (2007) have provided a comprehensive definition of the psychological capital as the positive psychological state of development which is characterized by hope, efficacy, resilience, and optimism. The theory of psychological capital focuses on how an individual is changing or evolving, rather than who that individual is. The four characterizing factors of psychological capital have been formed into an acronym “HERO.” The article by Youssef-Morgan and Luthans (2015) has described the four constituent characteristics as the key resources of psychological capital. Hope is defined as the positive motivational state of an individual which is based on the interactively derived sense of successful agency and pathways. From the organizational perspective, Cavus and Gokcen (2015) have mentioned that hope is the internalized willpower and determination of an individual to invest the energy to materialize organizational objectives. The hope of a leader influences the motivation, job satisfaction, and performance of the team members. It has been mentioned that hope supports the desire for positive outcomes and provides a feeling of emotional well-being. Hope has also been defined as a feature that drives people emotionally to voluntarily commit to their job roles and perform to the best of their abilities.

The second resource of psychological capital is efficacy. Youssef-Morgan and Luthans (2015) have defined efficacy as the personal belief of an individual to mobilize cognitive resources, motivation and courses of action to carry out a specific action in a particular context. It has been mentioned that there is a strong relationship between efficacy and individual performance. From the organizational perspective, Cavus and Gokcen (2015) have mentioned that efficacy, self-efficacy or confidence is the general belief of an individual which he/she perform in daily job roles. Team members with a high degree of self-confidence are able to improve their motivational levels, which is beneficial for their individual performance and eventually, for the performance of the organization as a whole. The authors have further mentioned that people with high self-efficacy often choose challenging tasks which pushes them to improve their performance while working against all obstacles to meet their goals. For an organization, working with team members with high efficacy can lead to higher competitive advantage, as it is able to undertake challenging tasks, thereby, generating more value for their stakeholders.

The third resource of psychological capital is resilience, which has been defined by the ability of an individual to rebound from conflict, adversity, and failure. It determines the reactive encounters of a challenge or a personal setback (Youssef-Morgan and Luthans, 2015). Cavus and Gokcen (2015) have mentioned in their article that the resiliency of an individual is his/her tendency to recover from a situation of negative emotional state and motivates to perceive an overwhelming situation in an optimistic manner. From the organizational perspective, it can be stated that individuals with high resiliency can overcome setbacks in their professional fields and look at the situation optimistically, rather than being demotivated by the situation. Thus, it is essential that employees should have a higher degree of resiliency in order to overcome negative emotional barriers, associated with professional setbacks. It also plays a crucial role in facilitating a seamless change management process. Resilient employees are least affected by the new changes brought in the operational processes and are likely to view the changes in a positive light. Thus, it can be stated that resiliency, as a part of psychological capital, can lead to better organizational performance, through higher engagement in their job roles.

The final resource of psychological capital is optimism. Youssef-Morgan and Luthans (2015) have defined optimism as a generalized positive outlook and expectancy of an individual toward external situations. Optimistic outlook defines positive events with the desirable outcome, in terms of permanent, personal and pervasive causes, whereas, the negative events are defined as situation-specific, external and temporary in nature. Just like Hope, Optimism has been explained as a dispositional trait and a state of mind that can be achieved or developed. Cavus and Gokcen (2015) have defined optimism in terms of the mental and physical health of an individual. It has been mentioned that optimistic people working in an organization are more likely to expect positive outcomes from any situation, which can lead to a reduction of work-induced stress and depression. Leaders with an optimistic attitude expect positive results from the subordinates, which, in turn, motivates them to work harder. Moreover, it also drives the employees to bear a positive outlook toward their job role and the organization as a whole.

A close look at the concept of psychological capital indicates that it allows employees in an organization to have a positive state of mind which enables them to perform at a superior degree. It creates a positive attitude toward work and fosters behavior among the employees, which leads to better organizational performance and higher competitive advantage. The article by Paterson, has described the psychological capital in light of ‘thriving at work.’ Thriving at work has been defined in terms of learning and vitality, both of which are essential for individuals to strive at in their respective organizations. These two factors can be achieved through higher psychological capital.

From the perspective of HRD, psychological capital plays a crucial role in organizational performance. This is especially applicable for entrepreneurs as a kind of strategic human resource, since psychological and cognitive elements of entrepreneurs could heavily determine entrepreneurial processes, decisions, and success (Baron, 2004, 2008; Baron and Ensley, 2006). For instance, Hmieleski and Baron (2008, 2009) indicated that both optimism and self-efficacy could significantly influence new venture success. Individuals with higher psychological capital are coincident about successful completion of their allocated task (efficacy), they are emotionally driven to pursue their goals and proactively plans for alternative ways to meet their objectives (hope), they are not affected by negative outcomes or professional setbacks in their day to day work (resiliency) and carries a positive outlook to every situation, irrespective of its outcome (optimism). Therefore, organizational leaders should ensure to create a work environment that can lead to higher psychological capital within the team members.

Paterson et al. (2014) have further mentioned that the behavior and attitude of an entrepreneur as an organizational leader can have a strong impact on the degree of psychological capital of an individual. It has been mentioned that supervisors or leaders who are able to create a supportive climate can foster higher psychological capital among the employees. Expressing concern about the well-being of the employees regarding their career development can drive them to push their own limits of individual performance. Moreover, working in a supportive climate can encourage employees to try new things and not be afraid of taking risks. Employees are more confident in their job role and are optimistic about the outcome of their actions. Moreover, the support of the leader also enables them to overcome negative outcomes and setbacks easily. Thus, supervisor support plays a crucial role in the development of human resources.

## Commentary

In the above sections, various articles have been reviewed, which have shed some light on the importance of psychological capital and its underlying resources. The discussion presented in this paper is in the context of entrepreneurial performance, competitive advantage, achieved by creating a positive mental state of the entrepreneurs. This article wishes to share some thoughts for the links between the PsyCap and HRD of entrepreneur (teams). Note, however, this article is not a comprehensive review of the literatures of PsyCap, HRD, and Entrepreneurship, which is nearly impossible to cover in a review article. On the contrary, the article focuses on the intersections of these key concepts and tried to shed some lights for future studies. Thus, is it also understandable that the article comes without an empirical part of a specific research question.

Psychological capital has been expressed in terms of the mental state of individuals and how it can positively influence their attitude toward their work and job role. It can be stated that the concept of psychological capital appropriately describes how the psychology and mental state of individuals determines their behavior and attitude at work. As mentioned previously, there are four key resources of psychological capital, such as hope, efficacy, resilience, and optimism. Each of these factors determines how individuals of entrepreneur teams or ventures perceive their job role and their relationship with their employer.

We think that a positive mindset of an employee regarding their work can prove to be beneficial for the organization. The employees are likely to have positive expectations from their employer, especially in terms of professional development and career prospects. As these expectations are met, the employees will have high hopes from their employer, which can motivate them to work harder and exhibit a higher degree of job engagement. We can also highlight that the hope of a leader or an entrepreneur can influence the mindset of an employee. Having high hopes from the team members emotionally drives them to materialize their objectives. Psychological capital is also defined in terms of the confidence of the employees. The confidence of an employee determines one’s ability to take up challenging tasks and complete them with ease. Highly confident employees are likely to have a strong impact on the ability of the organization to carry out challenging tasks, thereby, increasing its competitive advantage.

Amidst the steep competitive global business environment, it is essential for a firm to be able to differentiate itself by offering unique value to its customers and stakeholders. This can be achieved by working with entrepreneur teams with high confidence or efficacy. Psychological capital also determines the resilience of an individual and determines their ability to deal with setbacks and negative outcomes. We can discuss this aspect of psychological capital in the light of emotional intelligence. People with high emotional intelligence are more efficient in monitoring and regulating their own emotions (Petrides et al., 2016). Here, we can emphasize that employees with high resiliency factors are likely to have a high degree of emotional intelligence, as they are not easily affected by negative or undesired outcomes and even by uncertain situations. Employees who are largely unaffected by negative situations and are able to maintain a positive outlook on their work are more motivated to invest their cognitive resources into their job roles, leading to better performance. The final factor of psychological capital is optimism. As we have found, optimism is associated with the general perception of an individual toward a situation. An optimistic person is likely to have positive expectations from his/her work and job roles. Optimistic employees are motivated toward their job roles and are likely to work harder in their pursuit of professional development.

We can establish that psychological capital is defined as the mental or cognitive functions of an individual which determines their behavior and attitude toward the external environment, such as their profession. It has been found that psychological capital can be developed within an individual just like any other professional skills and abilities, which makes them more capable and efficient in their work. Therefore, it can be stated that organizational leaders seek to develop an entrepreneur team with a high degree of psychological capital, which can be beneficial to achieve better organizational performance and higher competitive advantage. It has been found that creating a supportive environment, where the leaders show genuine concern about the well-being and professional development of the team members can have a significant effect on improving their psychological capital.

From the works of literature, discussed in this paper, we can understand that, in order to achieve professional excellence, vocational skills and abilities are not the only underlying factors, that an entrepreneur should focus on. Developing and maintaining the right state of mind is also essential in determining how they are able to perform their daily activities. Apart from the skills and abilities of the employees, their willingness and motivation toward their work is also a crucial factor that entrepreneurs need to consider in the HRD activities.

## Future Research Opportunities

An in-depth assessment of psychological capital and its relationship with HRD has revealed that psychological capital can prove to be a crucial asset for an organization, which allows it to improve its performance and competitive advantage. Therefore, further studies are required to highlight how organizations can develop a team member with a high degree of psychological capital.

We have found that most of the existing studies are dedicated to defining and simplifying the concept of psychological capital and its importance for business organizations. The scholarly articles have explained the underlying resources of psychological capital but have failed to provide a deeper understanding of how organizations will be able to achieve it. We have learned from the articles that psychological capital can be developed just like any other skill, over time, thereby, necessitating the emphasis on training the employees, in order to ensure that the workforce has a high psychological capital.

Future research opportunities lie in the area of organizational training that can positively influence all four resources of psychological capital, which are hope, efficacy or self-confidence, resiliency, and optimism. Since psychological capital is associated with the behavioral and attitudinal aspects of the employees, training programs should be developed that specializes in those aspects. The study conducted by Owoyemi et al. (2011) has indicated that training programs can play a crucial role in improving the commitment level of the employees toward their work. Thus, training programs have been found to have a strong impact on moderating the behavior, attitude and perception of the employees. In this case of improving the psychological capital of the entrepreneur teams, it is essential for the firms to develop training programs that can improve upon all the four associated factors such as hope, efficacy, resilience, and optimism. In-depth research is required to find out how different aspects of training can influence each of the four factors as discussed previously.

Moreover, from the organizational perspectives, the importance of researching the cost-benefit ratio of such training programs can be highlighted. Almost all training programs, come with some degree of the cost that the organization needs to bear, in order to ensure that all the employees are prepared to meet their allocated job roles (Walker et al., 2016). In this case, before implementing new training programs dedicated to improving the psychological capital, the firms need to ensure that the positive benefits can outweigh the cost of running the training programs. New researches are required to empirically find out the commercial value of the training programs. The validity and reliability of the training programs need to be evaluated before they are implemented by the entrepreneur teams. The sole purpose of the training program is to improve the overall performance of the organization, eventually increasing the competitive advantage. Therefore, new researches that determine the economic validity of such training programs can prove to be helpful for organizations.

Future researches are also required to assess the effectiveness of the training programs on the team members with different personality traits. It has been found that psychological capital is associated with the behavior, attitude and perception of the individuals, therefore it is crucial to conduct an in-depth study about the personality traits which govern individual behaviors. As mentioned in the article by Matzler and Renzl (2007), different employees have different personality traits, which determine their behavior and response toward various external stimuli. Therefore, training programs which are designed to moderate the behavior of the employees can have a different impact on different employees, based on their personality traits. It can be further stated that based on the personality traits, different employees may react differently to their current organizational setting and may exhibit a different attitude toward their job role. Therefore, it is essential for the organization to selectively provide training to the employees who can benefit from it the most. Further researches are required to identify the potential candidates for the training program. New frameworks to determine the personality fit for the training programs should be developed. This can prove to be beneficial from the organizational stand point and also from the academic perspective. Future researches conducted in this area can help in developing new theories that can be applied to understanding the acceptance and effectiveness of behavior-moderating training among the employees.

Thus, it can be stated that entrepreneurs can be benefited by these future researches that determine the effectiveness of the training programs to improve psychological capital in the team members. The effectiveness of the training programs can only be ascertained if there are appropriate auditing methods that can identify significant changes in the organizational performance. Thus, new research is required to develop new auditing methods that can accurately measure the emotional state of an employee in a quantitative manner. It is essential to quantify the four identified resources of psychological capital, such as hope, efficacy, resiliency, and optimism. A theoretical framework that can quantitatively measure each of these factors for individual employees, can greatly help the organizational leaders and entrepreneurs to assess which area of psychological capital needs more emphasis. It can thus, help in implementing more effective training programs, which can have significant positive results. Furthermore, new measurement techniques need to be developed through further research which can help to identify the improvement in organizational performance by consequent increase in the four resources of psychological capital. Therefore, it is suggested that developing measurement and evaluation techniques by future research can help organizations to accurately measure the effectiveness of their initiatives to improve employee attitude and behavior.

The research suggested above can help to establish the relationship between psychological capital and HRD by entrepreneur teams and organizational leaders. In this context, the impact of national culture should also be explored. As mentioned in the study by Hofstede (2011), different nations have different cultural practices and they also determine the values, beliefs, and perceptions of its people. Therefore, we believe that employees from different cultural backgrounds will have different attitude toward their work and work environment. Employees with high scores in masculinity factor are likely to be more competitive in their work environment and employees with low score in individualism are likely to prefer working in teams. Thus, it can be stated that behavior and attitude of employees are likely to differ largely across national cultures. This, as a result, can make it quite challenging for entrepreneurs and organizational leaders to assess the mental state or the psychological capital of the entrepreneur teams. Thus, new research works are required to establish the impact of cultural background on the four resources of psychological capital. Moreover, establishing a relationship between different cultural dimensions and psychological capital will also enable organizations to design their training programs more efficiently. It is essential for firms to ensure that training programs are aligned with the cultural aspects of a nation, so as to improve the acceptance of the training program.

In the context of national culture and psychological capital, we should also highlight that achieving higher psychological capital in a diverse work environment can be challenging. As mentioned in the article by Saxena (2014), globalization has allowed organizations to hire employees from the global talent pool, which as a result has opened new possibilities in improving its competitive advantage. At the same time, it has also increased the cultural diversity of the team members, where employees from different national cultures, with different values, beliefs, and psychological profiles work together. Therefore, for the organizational leaders it can prove to be quite challenging to improve the psychological capital of the employees belonging to different cultural backgrounds. In order to address this issue, new research needs to be conducted in order to understand how cross-cultural management can be integrated within psychological capital. Entrepreneurial teams need to have a clear understanding of how a diverse employee base can be positively influenced in terms of their psychological capital. Moreover, developing and implementing a training program to improve the psychological capital of the diverse team can also become quite challenging. Since, different employees with varying values and beliefs are likely to have different responses and exhibit different degrees of acceptance toward the training programs, new researches are required to shed more light in this area. Further studies can help in formulating new cross-cultural management practices which can help in improving the psychological capital of the entire team members, even if it is culturally diverse in nature. Thus, it can be highlighted that more emphasis should be given on future research which focuses on workplace diversity. This should help the entrepreneurs develop their human resources in a globalized business environment.

The leadership practices within an organization can largely influence its performance and its corporate image. As mentioned in the article by Ahmad et al. (2014), leadership style followed in an organization can project a significant impact on the employee behavior, especially in terms of motivation and job engagement. The way in which an entrepreneur or an organizational leader interacts with the employees, devises organizational policies and encourages certain practices can have a prominent impact on the mental state of the employees, which can either drive them to voluntarily commit to organizational success or can also make them demotivated leading to increase in disengagement from their job roles. In this case, we can emphasize that future researches are required which are related to the leadership styles adopted by an entrepreneur and the degree of psychological capital of the team members. It is essential to find out how different leadership approaches can influence each of the psychological capital resources, which are hope, efficacy, resiliency and optimism. Different leadership styles can have different impact on an individual, based on their personality traits and psychological profile. Therefore, further research is required to determine how entrepreneurs can adopt different leadership styles to improve the psychological capital in the workplace.

Current researches have shown that psychological capital is associated with factors like hope, efficacy, resiliency and optimism. However, new research is required to identify various other factors which can have moderating effects on the psychological capital of the entrepreneurial teams. The identification of new moderating factors, can help the entrepreneur teams and organizational leaders to develop more effective HRD plans. It is also essential to conduct an in-depth study on various organizational factors that can have a prominent influence on the psychological capital within a firm. The study conducted by Anitha (2014) has highlighted how the working environment to which the employees are exposed on a daily basis can have a significant effect on their attitude and behavior toward their job role and fellow colleagues. In this context, it can be also stated that the factors associated with the work environment such as employee relationship, hygiene factors, interaction and engagement of the supervisors can influence the psychological capital of the employees. Thus, new study should be conducted in order to identify different work environmental factors and how they are responsible in influencing the psychological capital. This should help organizational leaders to create a work environment which can lead to better work performance. Moreover, it will also help to identify the negatively influencing factors which needs to be avoided. Thus, we can highlight the further research in the field of work environment and psychological capital should be empathized.

My commentary on psychological capital and its association with entrepreneur teams and HRD has revealed that the mental state or the psychological profile of an employee can have a strong impact on one’s attitude and behavior at work. Based on the in-depth review of various scholarly articles, we have found that psychological capital of an individual depends on four key factors which are hope, efficacy, resiliency and optimism. It plays a crucial role in determining the job engagement of the employees and their voluntary commitment toward their job roles. However, this area of study has significant potential for future researches. In this commentary we have looked at several suggestions for future researches, which can be beneficial for entrepreneurs and organizational leaders in order improve the psychological capital in their organization. Further studies can be conducted in the area of developing new training programs to improve psychological capital, its measurement, influence of cultural background, relationship with cross-cultural management, personality types, etc. We have also found that further study should be conducted to identify the impact of different leadership styles and work environment within an organization. These researches should help organizations to understand how the different aspects within an organization can have different impacts on the mental state or the psychological capital of the entrepreneur teams.

## Author Contributions

The author confirms being the sole contributor of this work and has approved it for publication.

## Conflict of Interest

The author declares that the research was conducted in the absence of any commercial or financial relationships that could be construed as a potential conflict of interest.
