# Correction: Psychological capital of entrepreneur teams and human resource development

**DOI:** 10.3389/fpsyg.2025.1693555

**Published:** 2025-09-22

**Authors:** Jun-Jun Tang

**Affiliations:** ^1^College of Economics and Administration, Nanjing University of Aeronautics and Astronautics, Nanjing, China; ^2^Huaiyin Institute of Technology, Huaian, China

**Keywords:** psychological capital, human resource development, future research, entreprenership, human capital

Affiliation Huaiyin Institute of Technology was erroneously omitted for author *Jun-Jun Tang*.

The original version of this article has been updated.

